# Volume-sensitive chloride channels are involved in cisplatin treatment of osteosarcoma

**DOI:** 10.3892/mmr.2014.3068

**Published:** 2014-12-09

**Authors:** SIYI CAI, TAO ZHANG, DANDAN ZHANG, GUIXING QIU, YONG LIU

**Affiliations:** 1 Department of Orthopedics, Peking Union Medical College Hospital, Chinese Academy of Medical Sciences and Peking Union Medical College, Beijing 100032, P.R. China; 2 Department of Internal Medicine, First Affiliated Hospital of Jinan University, Guangzhou, Guangdong 510630, P.R. China; 3 Department of Histology and Embryology, Medical College of Jinan University; Guangzhou, Guangdong 510632, P.R. China

**Keywords:** cisplatin, chloride channel, osteosarcoma

## Abstract

Chemotherapy is the most common therapeutic strategy used to treat osteosarcoma. The present study aimed to investigate the effects of functionally activated chloride channels on cisplatin-induced apoptosis of MG-63 human osteosarcoma cells. An MTT assay and flow cytometry were used to detect proliferation and apoptosis of the cells, respectively. Live cell imaging was used to detect volume changes in response to treatment with cisplatin and/or chloride channel blockers. The effects of these treatments on chloride currents were also assayed using the patch-clamp technique. The results of the present study indicate that chloride channel blockers may suppress cisplatin-induced apoptosis. The MG-63 cells cultured with cisplatin demonstrated an apoptotic volume decrease, as well as suppression of cell proliferation; which were reversed by co-treatment with chloride channel blockers. These results suggest that cisplatin may activate chloride channels, and that channel activation is an early signal in the pathways that lead to cisplatin-induced apoptosis and inhibition of proliferation in MG-63 cells. In conclusion, these results indicate that chloride channels have an important role in cisplatin treatment of osteosarcoma.

## Introduction

Osteosarcoma is the most common primary malignant bone tumor. A critical issue in the clinical therapy of osteosarcoma is the development of treatment strategies that kill cancer cells without harming normal healthy cells. (1). Cisplatin (*cis*-diamminedichloroplatinum II) is one of the most effective and widely used chemotherapeutic agents to treat solid tumors (2). Cisplatin causes DNA damage, which may lead to cell apoptosis; however, cisplatin treatment is often ineffective due to acquired drug resistance (3). Ion channels contribute to massive ion fluxes across plasma membranes and have important roles in diverse cell processes in healthy and disease states, including excitability, contraction, cell cycle regulation and metabolism, (4). Previous studies have suggested that chloride channels are associated with cell volume regulation, proliferation, cell cycle control and migration, as well as apoptosis (5–7). Apoptotic volume decrease (AVD) usually indicates cell apoptosis and is activated by ionic efflux, particularly of chloride ions, through volume regulatory anion channels (8). Ion channels have previously been proposed as potential targets for cancer therapy. A previous study suggested that treatment with a specific inhibitor of the cystic fibrosis transmembrane conductance regulator may be a novel therapeutic approach in the prevention of cisplatin-induced nephrotoxicity, without affecting the antitumor efficacy of cisplatin (9). Furthermore, impaired activity of volume-sensitive, outwardly rectifying (VSOR) chloride channels has been shown to contribute to the acquisition of cisplatin resistance in A549/CDDP cells (10). Conversely, Su *et al* (11) demonstrated that suppression of chloride channel 3 resulted in the inhibition of Akt and autophagy, which may enhance the therapeutic benefit of cisplatin in U251 human glioma cells. The present study aimed to investigate the role of chloride channels in cisplatin-induced apoptosis of MG-63 cells.

## Materials and methods

### Materials

All of the chemicals used in the present study were purchased from Sigma-Aldrich (St. Louis, MO, USA). The isotonic bath solution contained (in mM): 70 NaCl, 0.5 MgCl_2_, 2 CaCl_2_, 10 HEPES and 140 D-mannitol. The isosmotic solution was produced by replacing 70 mM NaCl with equimolar NaI, NaBr or sodium gluconate. The pipette solution consisted of (in mM): 70 N-methyl-D-glucamine chloride, 1.2 MgCl_2_, 10 HEPES, 1 EGTA, 140 D-mannitol and 2 ATP. Osmolarity of the solutions was detected using an automatic cryoscopic osmometer (Osmomat 030; Gonotec, Berlin, Germany). The pH of all bath and pipette solutions was adjusted to 7.4 and 7.25, respectively. The chloride channel blocker, 5-nitro-2-(3-phenylpropylamino)-benzoate (NPPB; 100 μmol/l; Sigma-Aldrich), was dissolved in dimethyl sulfoxide (DMSO; 100 mM; Sigma-Aldrich), and the other chloride channel blocker tamoxifen (20 μmol/l; Sigma-Aldrich) was dissolved in methanol anhydrous. NPPB and tamoxifen were diluted to final concentrations using isotonic solutions.

### Cell culture

The MG-63 human osteosarcoma cells (American Type Culture Collection, Manassas, VA, USA; no. CRL-1427) were cultured in Dulbecco’s modified Eagle’s medium (DMEM; Gibco Life Technologies, Carlsbad, CA, USA) supplemented with 10% fetal calf serum (FCS), 100 IU/ml penicillin and 100 μg/ml streptomycin (Sigma-Aldrich) in a humidified chamber containing 5% CO_2_ and 95% O_2_, at 37°C. The cells were collected at the logarithmic growth phase, resuspended, plated on coverslips and incubated for 1 h prior to further analysis.

### Chloride current recordings

Following stabilization of the background chloride current in isotonic solution, the bath solution was changed to isotonic solution containing 2 μg/ml cisplatin (CDDP) for 30–50 min. Once the cisplatin activated currents had reached their maximum, the bath solution was changed to cisplatin solution containing 100 μmol/l NPPB or 20 μmol/l tamoxifen for ~30min. Whole-cell Cl^−^ currents were recorded using the patch-clamp technique with 5–10 MΩ pipette resistance and an EPC-9 patch clamp amplifier (HEKA Electronik, Lambrecht/Pfalz, Germany). Whole-cell currents of individual cells were maintained at a constant voltage, then amplified and filtered at 2.9 kHz. The Cl^−^ equilibrium potential was set to 0 mV, then stepped to ±40 and ±80 mV for 200 ms repeatedly (12), with a 4 sec interval between pulses in voltage clamp mode, at 20–24°C. The currents were measured 10 msec after the onset of voltage steps. The background current was normalized in isotonic solution. The percentage of inhibition of the chloride channel blockers was calculated using the following equation: Inhibition (%)=[(C_CDDP_-C_Iso_)−(C_Blocker_-C_ISO_)]/(C_CDDP_-C_Iso_) × 100, where C_Iso_ is the background current under isotonic conditions; C_CDDP_ is the maximal stable current following exposure to cisplatin; and C_Blocker_ is the current recorded following treatment with the chloride channel inhibitors.

### Measurements of cell volume

Cells in the control group were incubated under isotonic conditions for 360 min. Cells in the treatment groups were incubated under isotonic conditions for 10 mins, then administered 2 μg/ml cisplatin alone or in combination with 20 μmol/l tamoxifen, and then incubated under isotonic conditions for a further 350 mins. Cells in the same field of view were imaged using an inverted phase contrast microscope (DMI6000 B; Leica Microsystems GmbH, Wetzlar, Germany) at 20–24°C, then analyzed using Scion software (Scion Corporation, Torrance, CA, USA) at the following time points: 0, 5, 10, 15, 20, 30, 40, 60, 80, 100, 140, 180, 220, 260, 300 and 360 min. The cell volume (V) was calculated from measured cell diameters (d) using the following equation: V=4/3π (d/2)^3^.

### Proliferation and apoptosis analysis

An MTT assay was used to detect the rate of cell proliferation. Cells were cultured in control medium (DMEM supplemented with FCS and antibiotics) or medium containing cisplatin (2 μg/ml) alone or in combination with 100 μmol/l NPPB or 20 μmol/l tamoxifen for 72 h. Cell suspensions containing 2.5×10^7^ cells/l were plated in 96-well culture plates, at a volume of 100 μl per well. The cells were incubated in normal media or media containing 2 μg/ml cisplatin and/or chloride channel blockers (NPPB; 100 μmol/l). MTT solution (10 μl/well; Sigma-Aldrich) was added to the cells, which were then incubated for 4 h prior to detection. The MTT solution was removed and replaced with DMSO, in order to dissolve the formazan crystals, for 15–30 min. The absorbance was then measured at a wavelength of 570 nm, using a Safire^2^ microplate reader equipped with the Magellan (5.0) reader software (Tecan Group, Ltd, Männedorf, Switzerland).

The rate of apoptosis of the cells was measured by flow cytometry. The cells were cultured for 72 h, (1–2×10^5^ cells/sample), then collected and analyzed. Briefly, the cells were washed three times with phosphate-buffered saline (PBS), fixed in chilled 70% ethanol at −20°C for 30 min, washed a further two times with PBS and incubated with RNase A (50 μg/ml in PBS) at 37°C for 30 min. The cells were then stained with propidium iodide (50 μg/ml; Beyotime, Shanghai, China) for 15 min and analyzed by flow cytometry (FACS101; BD Biosciences, Franklin Lakes, NJ, USA). The percentage of apoptotic cells was quantified using DNA histograms (FlowJo 7.6.1 software; FlowJo, LLC, Ashland, OR, USA).

### Statistical analysis

Data are expressed as the mean ± standard error. Significant differences were determined by analysis of variance using SPSS version 13.0 software (SPSS Inc., Chicago, IL, USA). P<0.05 was considered to indicate a statistically significant difference.

## Results

### Cisplatin-induced apoptosis can be suppressed by chloride channel blockers

**Figure 1:**
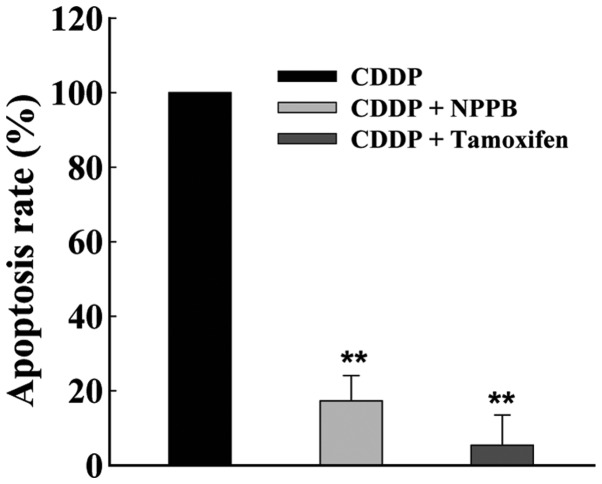
Chloride channel blockers decrease the rate of cisplatin-induced apoptosis in MG-63 human osteosarcoma cells. The cells were treated with cisplatin (CDDP; 2 μg/ml) in Dulbecco’s modified Eagle’s medium alone, or in combination with chloride channel blockers 5-nitro-2-(3-phenylpropylamino)-benzoate (NPPB; 100 μmol/l) or tamoxifen (20 μmol/l) for 72 h. The data represent the mean percentage of inhibition of apoptosis ± standard error of six experiments. ^**^P<0.01, vs. the control.

To determine the effects of chloride channels on apoptosis, MG-63 cells were cultured with cisplatin (2 μg/ml), either alone, or in combination with chloride channel blockers NPPB (100 μmol/l) and/or tamoxifen (20 μmol/l) for 72 h. The chloride channel blockers significantly suppressed the rate of cisplatin-induced apoptosis, as determined by flow cytometry. The rate of apoptosis inhibition was 84.7±15.8 and 94.4±18.1%, in response to treatment with NPPB and tamoxifen, respectively (n=6, P<0.01, Fig. 1).

### Chloride channel blockers prevent cisplatin-induced suppression of cell proliferation

**Figure 2:**
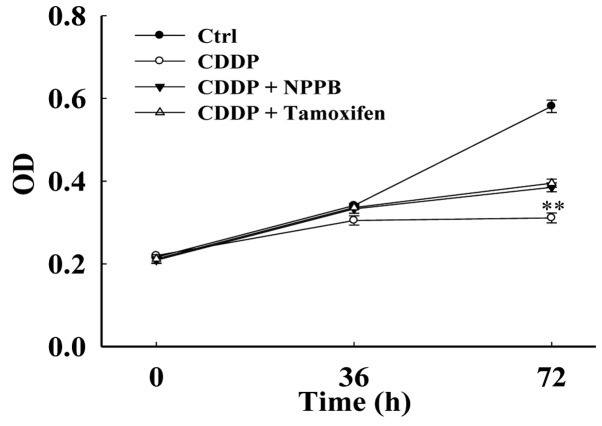
Chloride channel blockers increase proliferation of cisplatin-treated MG-63 human osteosarcoma cells. The cells were cultured in 96-well culture plates (at a density of 2,500 cells/well) in control medium overnight (~16–18 h), and were then incubated in control medium (Ctrl) or medium containing cisplatin (CDDP; 2 μg/ml) alone, or in combination with 5-nitro-2-(3-phenylpropylamino)-benzoate (NPPB; 100 μM) or tamoxifen (20 μM) for 36 or 72 h. The relative cell numbers were detected by an MTT assay and the optical density (OD) of the cells was measured. The figure shows the comparison of cell growth to standardized OD values at different time points in MG-63 cells. The data represent the mean ± standard error of three experiments. ^**^P<0.01, vs. the control.

The results of the present study indicate that chloride channel blockers may protect MG-63 cells against cisplatin-induced apoptosis. Therefore, the present study aimed to determine the effects of chloride channel blockers on the proliferation of cisplatin-treated MG-63 cells. The proliferation of MG-63 cells was evaluated by an MTT assay. The cells were cultured under the following four conditions: Control (DMEM), cisplatin (CDDP), CDDP+NPPB and CDDP+tamoxifen. The cells were cultured for 36 and 72 h, and the optical density of the cells was then measured using a microplate reader. The inhibition of proliferation was 77.0±23.5% following treatment with cisplatin for 72 h (Fig. 2). Treatment with NPPB and tamoxifen suppressed the cisplatin-induced inhibition of proliferation. The inhibition rate was reduced to 30.51±8.30% and 45.21±7.28%, in response to treatment with NPPB and tamoxifen, respectively (n=18, P<0.01).

### Chloride channel blockers inhibit cisplatin-induced AVD

**Figure 3:**
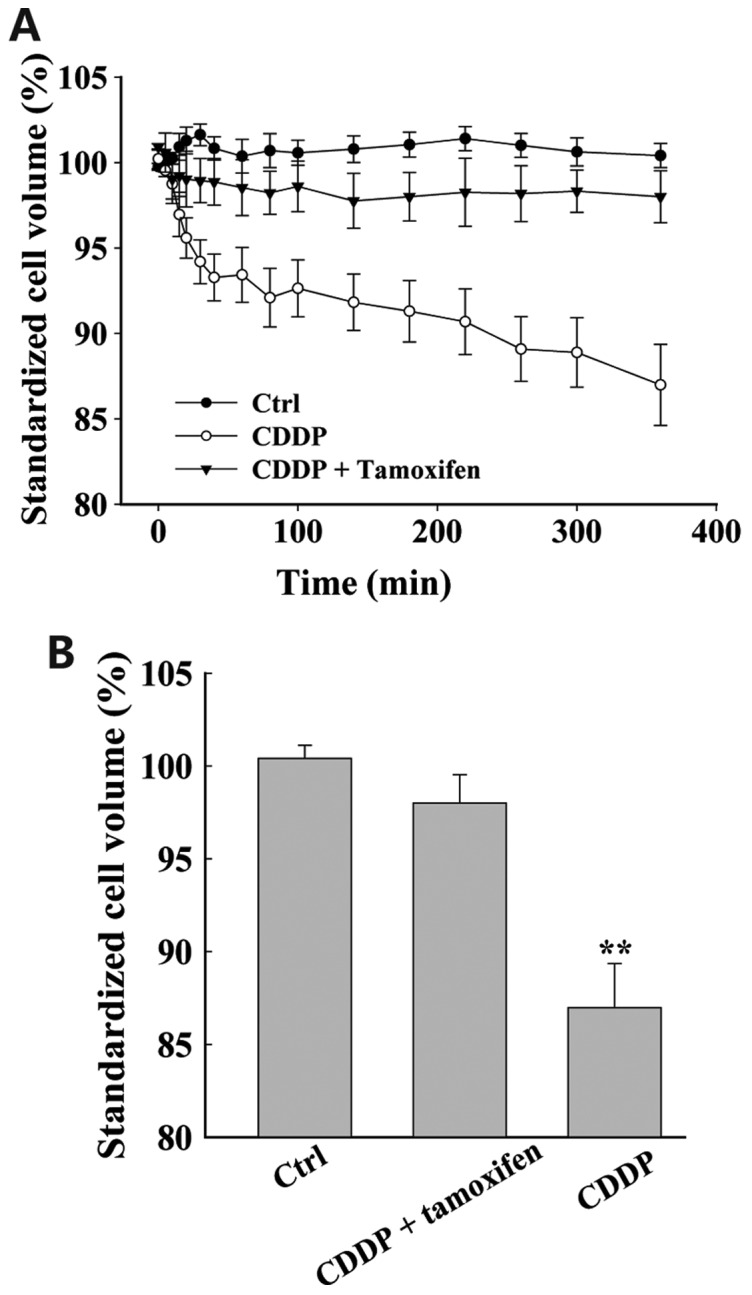
Chloride channel blocker tamoxifen prevents cisplatin-induced decreases in cell volume of MG-63 human osteosarcoma cells. (A) Time-dependent changes in MG-63 cell volume following incubation in isotonic bath solutions, with or without cisplatin (CDDP; 2 μg/ml) and tamoxifen (20 μM) treatment. (B) Final cell volumes following treatment for 360 min with different bath solutions. Cell volume analysis was standardized to that of the control group. The data represent the mean ± standard error of 23–32 imaged cells. ^**^P<0.01, vs. the control.

Live cell imaging was used to detect cell volume. Under isotonic conditions (control), the cell volume was stable (Fig. 3; n=18). However, when the cells were treated with cisplatin, cell volume gradually decreased. Cell shrinkage was detected as early as 10 min after application of cisplatin. After 6 h, cell volume had decreased by 15.1±2.2% (n=23, P<0.01). Cell shrinkage was alleviated in response to treatment with chloride channel blockers. Incubation with NPPB (data not shown) and tamoxifen suppressed the cisplatin-induced decrease in cell volume. In addition, the cell volume was not significantly different in the cells treated with chloride channel blockers, as compared with the control cells (n=32, P>0.05).

### Activation of chloride currents by extracellular application of cisplatin

**Figure 4:**
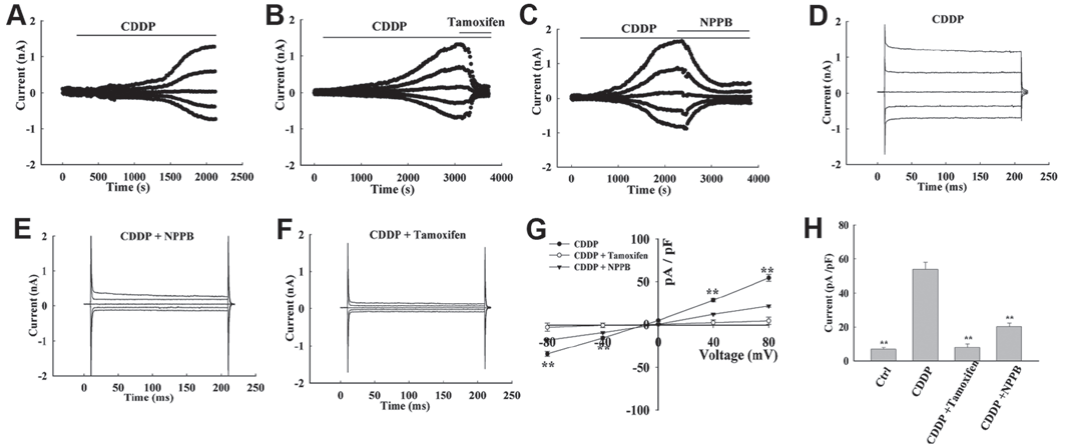
Cisplatin-induced chloride currents are inhibited by chloride channel blockers 5-nitro-2-(3-phenylpropylamino)-benzoate (NPPB) and tamoxifen in MG-63 human osteosarcoma cells. (A) Typical time course of the chloride current activated by cisplatin (2 μg/ml) in isoosmotic bath solution (CDDP) alone or in combination with chloride channel blockers (B) NPPB (100 μM) and (C) tamoxifen (20 μM). (D) Typical current traces recorded in the control CDDP bath solutions. Typical timecourse of inhibition of cisplatin-induced chloride currents by the chloride channel blockers (E) NPPB (100 μM) and (F) tamoxifen (20 μM). (G) Current-voltage relationships recorded in the control isotonic bath solution (Control) and in the cisplatin isoosmotic bath solution (CDDP) (mean ± standard error, *n*=15). (H) Chloride currents of control (Ctrl), cisplatin isoosmotic (CDDP) and cisplatin isoosmotic solutions containing NPPB or tamoxifen (mean ± standard error of 5–15 cells; ^**^P<0.01 vs. CDDP) at 80 mV. Cells were initially held at 0 mV, and voltage was then stepped to 0, ±40 and ±80 mV, repeatedly.

The results of the present study indicate that cisplatin may induce AVD, and it was hypothesized that chloride channels may be involved in this process. Whole cell patch-clamp recordings were taken, in order to determine the effects of cisplatin on chloride channel currents in MG-63 cells. The background chloride current in isotonic solution was weak and stable, with a density of 6.81±1.02 pA/pF at +80 mV; and −5.81±1.45 pA/pF at −80 mV (n=15, Fig. 4). In the majority of cells (7/10 cells), the currents were significantly increased in response to treatment with cisplatin for 10–15 min. The currents reached a plateau with mild outward-rectification at 30–50 min (Fig. 4D). The current displayed an almost linear current-voltage relationship, with an outward current of 53.96±4.01 pA/pF at +80 mV, and an inward current of −41.9±3.451 pA/pF at −80 mV (n=12, P<0.01, Fig. 4A). There was no time-dependent inactivation observed at ±40 and ±80 mV. The cisplatin-activated current reversed at −3.74±1.43 mV (n=15), which is close to the calculated Cl^−^ equilibrium potential (−0.9 mV). There was no potassium shown to be present in either the pipette or bath solutions. In addition, equilibrium potentials for Na^+^ and Ca^2+^ were predicted to be >+200 mV. Therefore, these results support the conclusion that the cisplatin-activated current is generated primarily by Cl^−^.

### Chloride channel blockers inhibit cisplatin-activated chloride currents

Once the cisplatin-activated currents had reached the maximum, the bath solution was changed to cisplatin solution containing NPPB and tamoxifen. Extracellular application of NPPB and tamoxifen inhibited the current (Fig. 4B and C). The outward and inward currents were almost equally inhibited, and the inhibition rate was 70.21±3.08% and 97.88±1.50% at +80 mV, and 68.321±4.98% and 98.36±2.04% at −80 mV, respectively (n=5, Fig. 4G and H). In these experiments, DMSO was used to prepare the NPPB solutions, and the final concentration of DMSO in the bath solutions did not exceed 0.1% (v/v). At this concentration, it did not affect cell volume or current, and was not cytotoxic within the 24 h period of analysis.

### Anion selectivity of the cisplatin-activated chloride channel

**Figure 5:**
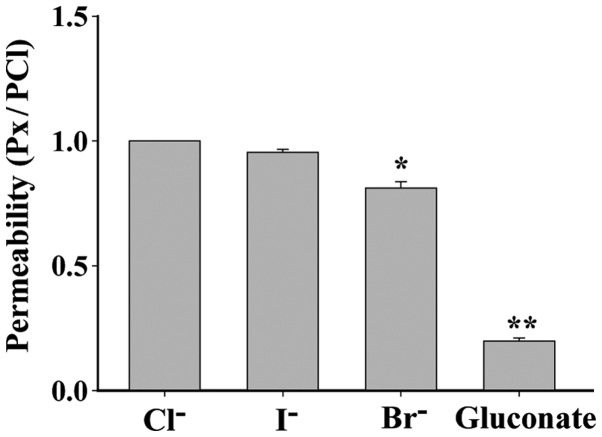
Anion permeability of the cisplatin-activated chloride channel. Anion selectivity of the cisplatin-activated chloride channel was determined by replacing the 70 mM NaCl of the isotonic solution with equimolar Na (X), where X represents the substituted anion I^−^, Br^−^ or gluconate. The anion permeability, relative to that of Cl^−^, was calculated from the shift in reverse potential. The order of anion permeability was Cl^−^=I^−^> Br^−^>gluconate acid ion. The data represent the mean ± standard error of six experiments. ^*^P<0.05 and ^**^P<0.01, vs. Cl^−^.

Anion selectivity of the cisplatin-activated current was determined by replacing 70 mM NaCl with equimolar Na (X), where X represents the substituted anion I^−^, Br^−^, or gluconate. The permeability of I^−^, Br^−^ and gluconic acid ion, as compared with Cl^−^, which was defined as 1, was: I^−^, 0.95±0.01 (P>0.05, n=5); Br^−^, 0.81±0.03 (P<0.05, n=6), gluconic acid ion, 0.20±0.01 (P<0.01, n=6). Therefore, the order of the anion permeability of the cisplatin-activated channel was Cl^−^= I^−^>Br^−^>gluconate acid ion (Fig. 5).

## Discussion

Osteosarcoma is the most common primary malignant bone tumor in children and adolescents. There are numerous chemotherapeutic drugs that exert different pharmacological effects; however, the current obstacle to clinical chemotherapy is the development of treatment strategies that kill tumor cells, without harming normal healthy cells (1). Cisplatin is often used as a single agent, or in combination with other drugs, and is effective in the treatment of various types of tumor (13).

Cisplatin is a platinum complex that exhibits profound cytotoxic effects. It has a strong broad-spectrum anticancer effect, and is the most common drug used to treat nasopharyngeal carcinoma, osteosarcoma and other solid tumors (14,15). Cisplatin can damage DNA and cause apoptosis, and is capable of acting synergistically with various chemotherapeutic drugs to increase effectiveness; however, the mechanisms by which this occurs are not fully understood. Furthermore, cytotoxicity and drug resistance to cisplatin reduce its effectiveness. Understanding how cisplatin exerts its effects is crucial in the development of improved treatment strategies. The present study hypothesized that cisplatin may exert its functions through chloride channels.

Chloride channels are widely distributed throughout mammalian tissues. In addition to regulating cell volume, volume-sensitive chloride channels have been shown to correlate with cell cycle regulation, and proliferation, migration and apoptosis of cells (4,16–22). AVD marks the early stages of apoptosis, and is closely associated with the activation of chloride channels (23). The present study explored the role of chloride channels in cisplatin treatment of osteosarcoma, with the aim of identifying novel targets for osteosarcoma therapy.

The present study examined the role of chloride channels in cisplatin-induced apoptosis of MG-63 cells. Flow cytometry and an MTT assay were used to detect the rate of apoptosis and proliferation, respectively. In the cells treated with cisplatin, cell proliferation was suppressed and the rate of apoptosis was increased. Furthermore, co-treatment of cisplatin with chloride channel blockers NPPB and tamoxifen resulted in a reduction in the rate of cell apoptosis and inhibition of cell proliferation, which had initially been induced by cisplatin. These results suggest that chloride channels are activated and involved in cisplatin-induced apoptosis.

To demonstrate that activation of chloride channels is a key signal controlling the early stages of apoptosis, live cell imaging and whole cell patch-clamp recordings were used to detect cisplatin-induced changes in cell volume and chloride currents, in osteosarcoma cells. Live cell imaging analysis showed that cell volume gradually decreased in response to treatment with cisplatin; this type of continuous cell volume decrease has previously been shown to activate apoptosis (24,25). Whole cell patch-clamp analysis indicated that chloride channel currents were significantly increased upon treatment with cisplatin, and that the cisplatin-activated current was reversed at a potential close to the calculated chloride equilibrium potential (−0.9 mV). The typical current traces indicate that the currents were not time-dependent. In addition, chloride channel blockers were shown to inhibit cisplatin-activated chloride currents. Further experiments were then conducted to determine anion selectivity of the channel, and the order of anion permeability was shown to be Cl^−^=I^−^ >Br^−^>gluconate acid anion. These results suggest that cisplatin activates chloride channels, opening them and thereby driving the flow of chloride ions out of the cell, causing the cells to shrink, and thus resulting in a decrease in cell volume and ultimately induction of cell apoptosis (26,27).

It is possible that other ion channels may also be involved in osteosarcoma therapy. A previous study showed that capacitative Ca^2+^ entry influx may activate transient receptor potential channels, which may affect cell proliferation of osteosarcoma cells (28). Kv1.3 is another channel that may be involved in the treatment of osteosarcoma. Previous research demonstrated that a knockdown of Kv1.3 significantly inhibited the growth of MG-63 xenografts (29). In addition, other groups have reported that treatment with trichostatin A can restore functional expression of VSOR chloride channels, and that this may lead to a decrease in the cisplatin resistance of KCP-4 human epidermoid cancer cells. These results suggests that impaired activity of VSOR chloride channels may be involved in the acquisition of cisplatin resistance in this type of cancer (30). Ransom *et al* (31) demonstrated that glioma cell migration through brain tissue may require volume-activated chloride currents, which participate in cell shape and volume changes. Furthermore, tumor necrosis factor-mediated liver cell death has been shown to be triggered by the activation of K^+^ and Cl^−^ channels, which is an early signal in apoptotic pathways (32). These results further demonstrate that numerous types of ion channels, particularly chloride channels, are involved in tumor growth, apoptosis and migration.

The results of the present study demonstrate that cisplatin activates chloride channels, causing a cell volume decrease, which may lead to apoptosis of osteosarcoma cells (26,27). It may therefore be concluded that chloride channel activation is an early signal in pathways leading to cisplatin-mediated suppression of proliferation and induction of apoptosis in MG-63 cells. Identification of the specific chloride channel activated by cisplatin may be a potential focus of future research.
